# Generation of in situ CRISPR-mediated primary and metastatic cancer from monkey liver

**DOI:** 10.1038/s41392-021-00799-7

**Published:** 2021-12-03

**Authors:** Liping Zhong, Yong Huang, Jian He, Nuo Yang, Banghao Xu, Yun Ma, Junjie Liu, Chao Tang, Chengpiao Luo, Pan Wu, Zongqiang Lai, Yu Huo, Tao Lu, Dongni Huang, Wenlin Gong, Lu Gan, Yiqun Luo, Zhikun Zhang, Xiyu Liu, Yongxiang Zhao

**Affiliations:** 1 National Center for International Research of Biotargeting Theranostics, Guangxi Key Laboratory of Biotargeting Theranostics, Guangxi Medical University, Nanning, Guangxi 530021 China; 2 Department of Hepatobiliary Surgery, The First Affiliated Hospital, Guangxi Medical University, Nanning, Guangxi 530021 China; 3 Department of Pathology, Tumor Hospital, Guangxi Medical University, Nanning, Guangxi 530021 China; 4 Department of Ultrasound Imaging, Tumor Hospital, Guangxi Medical University, Nanning, Guangxi 530021 China

**Keywords:** Cancer models, Cancer models

## Abstract

Non-human primates (NHPs) represent the most valuable animals for drug discovery. However, the current main challenge remains that the NHP has not yet been used to develop an efficient translational medicine platform simulating human diseases, such as cancer. This study generated an in situ gene-editing approach to induce efficient loss-of-function mutations of *Pten* and *p53* genes for rapid modeling primary and metastatic liver tumors using the CRISPR/Cas9 in the adult cynomolgus monkey. Under ultrasound guidance, the CRISPR/Cas9 was injected into the cynomolgus monkey liver through the intrahepatic portal vein. The results showed that the ultrasound-guided CRISPR/Cas9 resulted in indels of the *Pten* and *p53* genes in seven out of eight monkeys. The best mutation efficiencies for *Pten* and *p53* were up to 74.71% and 74.68%, respectively. Furthermore, the morbidity of primary and extensively metastatic (lung, spleen, lymph nodes) hepatoma in CRISPR-treated monkeys was 87.5%. The ultrasound-guided CRISPR system could have great potential to successfully pursue the desired target genes, thereby reducing possible side effects associated with hitting non-specific off-target genes, and significantly increasing more efficiency as well as higher specificity of in situ gene editing in vivo, which holds promise as a powerful, yet feasible tool, to edit disease genes to build corresponding human disease models in adult NHPs and to greatly accelerate the discovery of new drugs and save economic costs.

## Introduction

As well all know, the current success rate of new drug discovery is very low (<10%). The main reason for this challenge is that no ideal animal model mimicking human diseases (such as cancer) is available. Experimental animal models, such as rodents and non-human primates (NHPs), are essential for both cancer research and pre-clinical assessment of anti-tumor drugs and vaccines.^1,2^ Of the two models, rodents are more widely used because of a number of advantages, such as rapid propagation, small size, low cost, clear genetic background, and availability of transgenic techniques. However, a large proportion of anti-tumor drugs, which have shown effectiveness in mice and rats, failed to achieve a similar efficacy or safety in human clinical trials, which has been mainly attributed to marked species differences between humans and rodents.^3,4^ NHPs, such as the cynomolgus monkey and African green monkey, exhibit considerable similarities, including genetic, physiological, and immunological characteristics, to humans. Thus, NHPs represent more superior animal models compared to rodent or other animal models used for disease research, efficacy and safety assessment of drugs, and testing of new therapeutic approaches.^5,6^ In fact, nearly all known human cancer genes have also been identified in monkeys and chimpanzees. Currently, mouse models of cancer are usually created using gene targeting in embryonic stem cells or somatic cell nuclear transfer methods, through which knock-out, knock-in, and precise modifications of the desired genes are achieved.^7^ Despite their apparent advantages, the use of experimental NHPs in cancer research and pre-clinical evaluation of anti-tumor drugs have been very limited, which is largely due to high costs and serious ethical concerns. In addition, compared to mice and rats, creating loss-of-function or gain-of-function genetic mutations through the breeding of NHPs remains tedious and challenging because of their longer sexual maturation time and slower propagation compared to rodents or other small model organisms.^7,8^

Recently, an emerging genomic editing technology, known as Clustered Regularly Interspaced Short Palindromic Repeats (CRISPR)/CRISPR-associated nuclease 9 (Cas9) (CRISPR/Cas9), has been developed and dramatic progress has been made in its application to research. CRISPR/Cas9-mediated site-specific DNA double-strand breaks, which are considered key lesions to activate two different intrinsic DNA repair mechanisms, i.e., non-homologous end joining (NHEJ) and homology-directed repair (HDR), can be repaired by either the HDR or NHEJ pathway, thereby allowing the specific gene to be edited.^9–13^ The CRISPR/Cas9 system, originally recognized as an adaptive immune defense mechanism in bacteria, is composed of the Cas9 protein as a nuclease and the single-guide RNA (sgRNA) as its two major components. With the guidance of sgRNAs, the Cas9 protein is recruited to the target site to cut the DNA, allowing removal (deletion) and addition (insertion) of DNA fragments.^14,15^ Indeed, the CRISPR/Cas9 system is a revolutionary, yet feasible genome-editing tool. It has been successfully and effectively applied in rodent and NHP embryonic stem cells and zygotes, and also in a broad range of organisms including plants, rodents, and other small experimental animals.^16–21^ But, until now, in situ adult monkey gene-edited primary liver cancer has not, to the best of our knowledge, been reported, and in situ CRISPR-mediated metastatic hepatoma in adult monkeys is even more unimaginable.

In the present study, the *Pten* and *p53* genes, two well-known and proven tumor suppressor genes,^22–25^ were selected as two target genes to produce mutations and to create cynomolgus monkey models of human primary and metastatic liver cancer. Our goal was to deliver the CRISPR/Cas9 system into the liver of cynomolgus monkeys through intrahepatic portal vein injection under ultrasound guidance, and to assess its potential to rapidly generate NHP models with somatic genetic mutations that more closely mimic human primary and metastatic liver cancer.

## Results

### Editing efficiency of the CRISPR/Cas9 system for the *Pten* and *p53* genes in COS-7 monkey cell line

To create genomic loss-of-function mutations in the tumor suppressor genes *Pten* and *p53* using the CRISPR/Cas9 genomic editing system, we constructed Adeno-Cas9 capable of expressing the Cas9 protein (Supplementary Fig. 1a), Adeno-Cherry-sgRNA capable of expressing mCherry protein (Supplementary Fig. 1b), Adeno-Pten-sgRNA and Adeno-p53-sgRNA capable of expressing the corresponding sgRNAs. Adeno-Pten- sgRNA vector was designed using 1 sgRNA with the N20NGG pattern to target mutation G129E of the first exon in the *Pten* gene (Supplementary Fig. 1c). Adeno-p53-sgRNA vector was designed using the N20NGG pattern to simultaneously target mutation R248G and R249G of the first exon in the *p53* gene (Supplementary Fig. 1d). In parallel, the Adeno-Cherry-sgRNA was generated as a control expression vector using the parent vector, adenovirus, and sgRNA targeting the red fluorescent protein (Cherry) gene (Supplementary Table 1).

Prior to the investigation of the potential for intrahepatic portal vein injections of the CRISPR/Cas9 system to generate genomic loss-of-function mutations of the *Pten* and *p53* genes in the cynomolgus monkey livers, we separately examined the editing efficiency of Adeno-Pten-sgRNA and Adeno-p53-sgRNA on the *Pten* and *p53* genes in the COS-7 cell line. The COS-7 cells were transiently transfected with Adeno-Pten-sgRNA and Adeno-cas9, Adeno-p53-sgRNA, and Adeno-cas9 for 48 hr, and total genomic DNA was extracted and the *Pten* and *p53* loci were analyzed by the T7E1 assay. The T7E1 digestion generated two fragments as expected (Supplementary Fig. 2a, c). Transfection of Adeno-Pten-sgRNA resulted in a significant induction of 22 indels in 30 clones, of which sixteen were deletions denoted by dotted blue lines, whereas six were an insertion in the *Pten* gene as indicated by red capital letters (Supplementary Fig. 2b). Transfection of Adeno-p53-sgRNA resulted in 20 indels in 30 clones, of which 15 were deletions denoted by dotted blue lines, whereas five were an insertion in the *p53* gene as indicated by red capital letters (Supplementary Fig. 2d). As illustrated in Supplementary Fig. 2b for the representative indel sequences in the *Pten* gene, the deletions ranged from 2 to 16 bp in length, whereas insertions from 3 to 8 bp were induced. Supplementary Fig. 2d for the representative indel sequences in the *p53* gene, the deletions ranged from 6 to 15 bp in length, whereas the insertions were from 1 to 4 bp. The efficiency of the indels induced by the CRISPR/Cas9 system for genomic editing of the *Pten* and *p53* genes were 73.33% (22 out of 30 colonies) and 66.67% (20 out of 30 colonies) in COS-7 cells, respectively.

### Superiority of intrahepatic portal vein injection of CRISPR/Cas9 system guided by color ultrasound

**Fig. 1:**
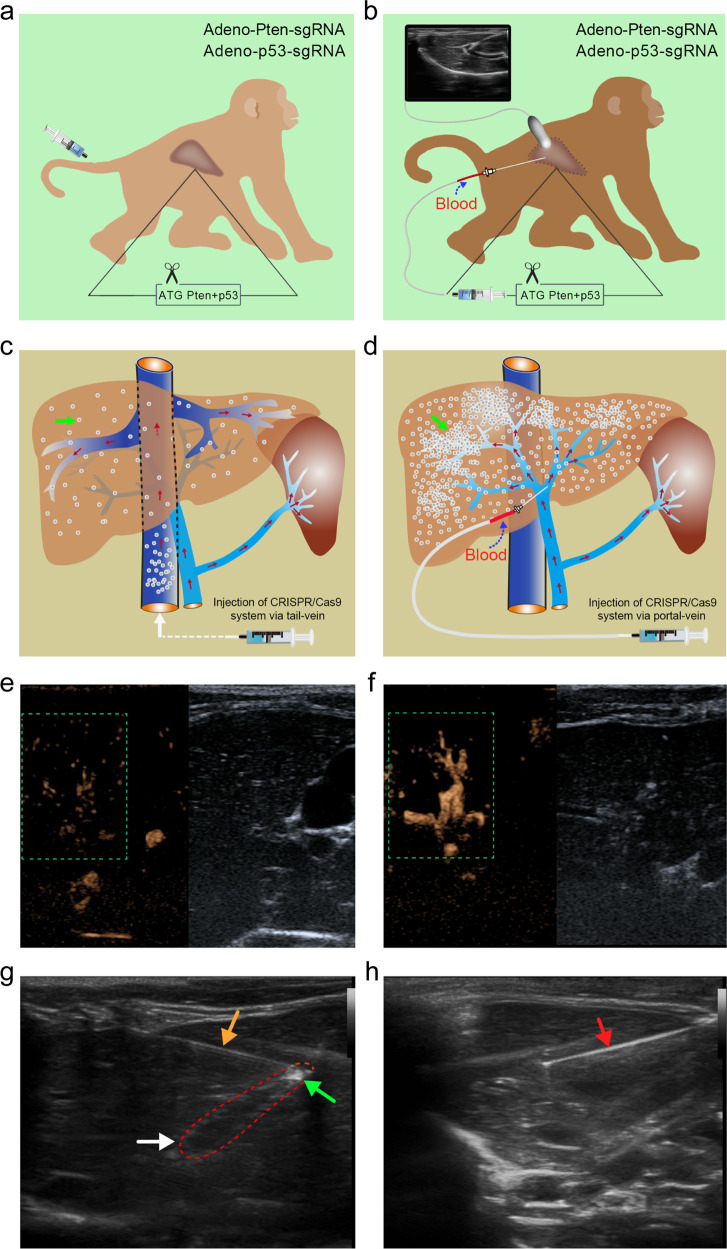
Intrahepatic portal vein color ultrasound-guided CRISPR/Cas9 system for *Pten* and *p53* genes into cynomolgus monkey livers. **a** The diagram of tail vein injection site under sedation. **b** The diagram of color ultrasound-guided intrahepatic portal vein injection site under sedation. **c** The diagram of the diffusion of the color ultrasound contrast agent via tail vein injection. **d** The diagram of the diffusion of the color ultrasound contrast agent into cynomolgus monkeys via intrahepatic portal vein injection guided by color ultrasound. The color ultrasound contrast agent is denoted by a green arrow in **c** and **d**. **e** and **f** are composed of the ultrasound imaging of liver vascular (the left) and the two-dimensional ultrasound imaging (the right). The concentrations of contrast agents in liver tissue are indicated by the green box. **g** Images of injection of the CRISPR/Cas9 system by Percutaneous Ethanol Injection Therapy (PEIT) needle through the intrahepatic portal vein under the ultrasound guidance. The needle is denoted by a yellow arrow, the sagittal section of the intrahepatic portal vein is marked by a white arrow, and the CRISPR/Cas9 expression vector delivered by intrahepatic portal vein injection is indicated by a green arrow. **h** Imaging of percutaneous liver biopsies from the right lobe of the liver. The needle is denoted by a red arrow

In order to choose an ideal way to deliver the CRISPR/Cas9 system into in vivo liver tissue, we compared the times for the ultrasound contrast agent to reach the liver, the times to peak concentration, and the retention times in the liver after the intrahepatic portal vein injection and the tail vein injection of the contrast agent. (Fig. 1a, b). In the ultrasound-guided intrahepatic portal vein injection group, the time of the drug (contrast agent) reaching the liver was very short, only 3 sec, the peak time of maximum concentration appeared high at 7th second (see green box in Fig. 1f), and the retention time lasted 59 sec (Fig. 1d, f), and the concentration in liver tissue was high. However, in the tail vein injection group, the time for the drug to reach the liver was 7 sec, the peak time of maximum concentration showed low at 14th second (see green box in Fig. 1e), and the dissipation time was 20 sec (Fig. 1c, e). The concentration of liver tissue (see green box in Fig. 1e) was low. Compared with the tail vein injection, the time of peak drug concentration using intrahepatic portal vein administration guided by color ultrasound were markedly earlier, the maximum drug concentration remarkably increased, and the retention time of the drug in the liver tissue significantly prolonged, which was two times more than that of tail vein injection. Therefore, we thought that color ultrasound-guided intrahepatic portal vein injection to deliver the CRISPR/Cas9 system was more superior (Supplementary Video 2, 3).

### In situ mutation of *Pten* and *p53* genes using intrahepatic portal vein ultrasound-guided CRISPR injection into adult monkey livers

**Fig. 2:**
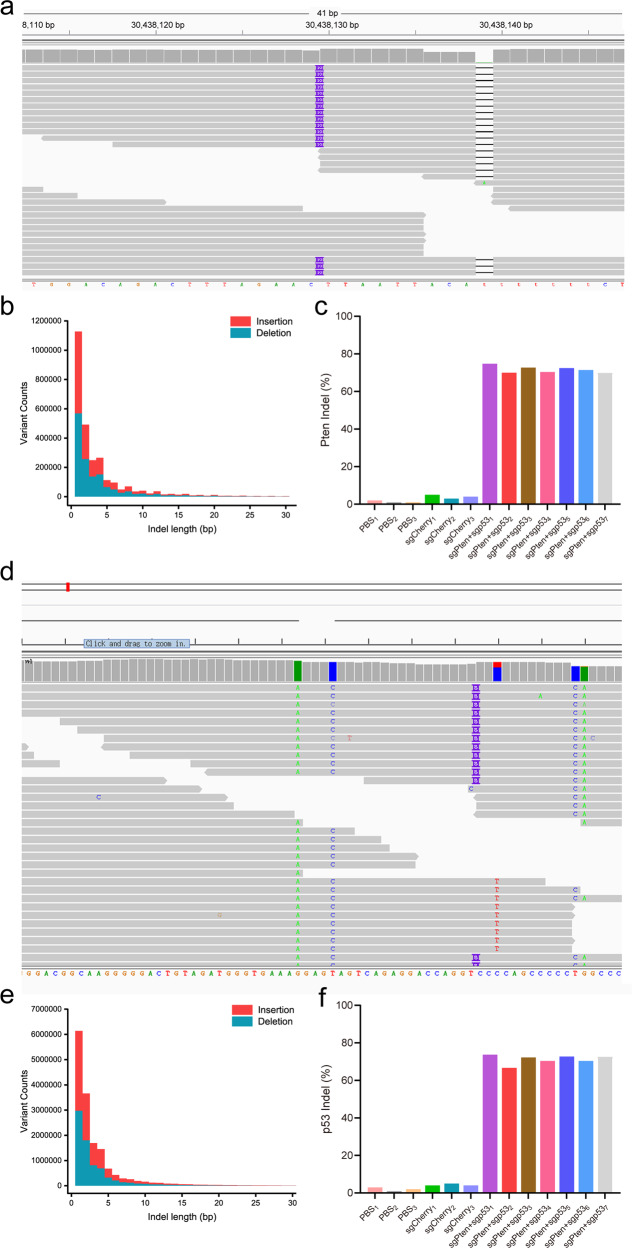
WGS of the *Pten* and *p53* genes using the CRISPR/Cas9 system in cynomolgus monkeys. **a** WGS analysis of the *Pten* locus in a representative mutant cynomolgus monkey when the tumor masses were found on ultrasound ~7–10 months later after delivery of the CRISPR/Cas9 system. The deletions are denoted by black lines and insertions are indicated by purple lines. **b** Distribution of indel length of the *Pten* locus in a representative mutant cynomolgus monkey when the tumor masses were found on ultrasound ~7–10 months later after delivery of the CRISPR/Cas9 system. Blue represented deletion and red represented insertion. The results showed that the indel length varied from 1–20 bp and deletion was dominant. **c** The frequency of *Pten* gene indels in the PBS-treated cynomolgus monkeys (*n* = 3), sgCherry-treated cynomolgus monkeys (*n* = 3), and sgPten+sgp53 indel cynomolgus monkeys (*n* = 7). **d** WGS analysis of the *p53* locus in a representative mutant cynomolgus monkey when the tumor masses were found on ultrasound ~7–10 months later after delivery of the CRISPR/Cas9 system. The deletions are denoted by black lines and insertions are indicated by purple lines. **e** Distribution of indel length of the *p53* locus in a representative mutant cynomolgus monkey when the tumor masses were found on ultrasound ~7–10 months later after delivery of the CRISPR/Cas9 system. Blue represented deletion and red represented insertion. The results showed that the indel length varied from 1–20 bp and deletion was dominant. **f** The frequency of *p53* gene indels in the PBS-treated cynomolgus monkeys (*n* = 3), sgCherry-treated cynomolgus monkeys (*n* = 3), and sgPten+sgp53 indel cynomolgus monkeys (*n* = 7)

We next examined whether intrahepatic portal vein injections of the CRISPR/Cas9 system could effectively deliver the Adeno-Pten-sgRNA and Adeno-p53-sgRNA into the livers of cynomolgus monkeys for genomic editing of the *Pten* and *p53* genes in order to obtain loss-of-function effects. Fourteen cynomolgus monkeys were anesthetized and the sagittal section of the left intrahepatic portal vein was located under ultrasonic guidance. Then, the CRISPR/Cas9 system with Adeno-Pten-sgRNA and Adeno-p53-sgRNA in normal saline was rapidly injected into the liver of each monkey once every 2 weeks for a total of 4–6 times. (Fig. 1g). Meanwhile, the equal amount of the Adeno- Cherry-sgRNA and PBS were delivered as two control experiments. When the tumor masses were found on ultrasound ~7–10 months later, liver biopsies were obtained from cynomolgus monkeys (Fig. 1h) and total genomic DNA was isolated for whole-genome sequencing (WGS). WGS and characterization of the *Pten* and *p53* gene loci in each cynomolgus monkey indicated that intrahepatic portal vein injections of Adeno-Pten-sgRNA and Adeno-p53-sgRNA led to induction of indels for the *Pten* and *p53* genes in seven out of eight experimental cynomolgus monkeys (87.5%). Further analysis of WGS revealed both deletions denoted by black lines and insertions indicated by purple lines, ranging from 1 to 20 bp in length (Fig. 2a, d). The frequencies of the indels mediated by the CRISPR/Cas9 system for genomic editing of the *Pten* gene in the sgPten + sgp53-1 to sgPten+ sgp53-7 were 74.71%, 72.73%, 72.41%, 71.43%, 70.33%, 70.00% and 70.00%, respectively, and the mean frequency was 71.56% (Fig. 2b, c); the p53 gene in the sgPten+sgp53-1 to sgPten+sgp53-7 were 74.68%, 72.73%, 72.22%, 70.37%, 72.73%, 70.37%, and 66.67%, respectively, and the mean frequency was 71.40% (Fig. 2e, f). Moreover, no mutant genes were observed in sgCherry or PBS group. These data suggested that delivery of Adeno-Pten-sgRNA and Adeno-p53-sgRNA into the livers of the cynomolgus monkeys through intrahepatic portal vein injection generated the putative loss-of-function mutations in the *Pten* and *p53* genes.

### Color ultrasound imaging of liver lesions following intrahepatic portal vein ultrasound-guided CRISPR injection into monkey livers

**Fig. 3:**
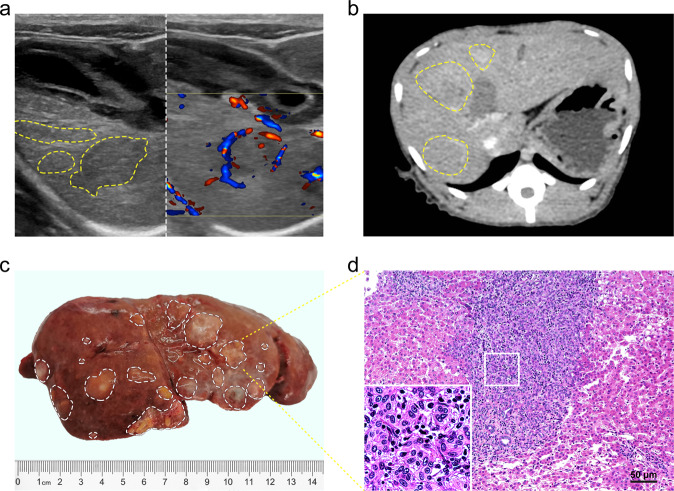
Modeling primary multiple liver cancers in cynomolgus monkeys using the CRISPR/Cas9 system. **a** Representative of ultrasound images of liver cancer cynomolgus monkeys. The ultrasound showed that multiple hypoechoic membranes of varying sizes were shown on the two-dimensional ultrasound image to surround the entire cancerous tumor, the capsule was intact, and the internal echo was different. The two-dimensional ultrasound detected multiple heterogeneous echoes in the right lobe of the liver. The tumor diameter is between 0.7~2.3 cm (the left). Color Doppler Flow Imaging showed color inlaid “cluster” plaques, the surrounding blood flow appeared as a full circle or arc surrounding (the right). **b** Representative of liver CT scan images of liver cancer cynomolgus monkeys. The tumor diameter is between 1.3~2.7 cm. **c** The cynomolgus monkeys were anesthetized and euthanized, and the livers were removed for HE and IHC staining when the tumor sizes were >2.0 cm in diameter on the ultrasound. Representatives cynomolgus monkeys displayed many visible liver tumor nodules of different sizes in the liver. **d** HE staining confirmed hepatocellular carcinoma. The insets were high-magnification views (×1000)

When the tumor sizes were >2.0 cm in diameter on the color ultrasound, the ultrasound imaging showed the liver surface was uneven, with multiple irregular-shaped mass echo images, 0.7–2.3 cm in diameter, unclear boundary, low echo, and less uniform (the left of Fig. 3a, Supplementary Fig. 4d). Color Doppler flow imaging (CDFI): the tumor was probed and rich in color blood flow signals with bright colors (the right of Fig. 3a). Contrast-enhanced ultrasound: In the arterial phase, the ultrasound contrast agent filled up quickly and showed high enhancement, whereas in the late portal or venous phase, the ultrasound contrast agent disappeared quickly and displayed low enhancement. The overall tumor demonstrated the malignant characteristics of rapid filling and disappearing. (Supplementary Video 1).

### CT imaging of liver lesions following intrahepatic portal vein ultrasound-guided CRISPR injection into monkey livers

When the tumor sizes were >2.0 cm in diameter on the ultrasound, the CT imaging showed that multiple low-density lesions, uniform density, irregular shape, unclear edges, blurred liver parenchyma boundaries, soft tissue shadow blocks, ~1.3–2.7 cm in diameter (see yellow circles in Fig. 3b). Obviously, uneven enhancement was seen in the arterial enhancement phase, whereas the enhancement degree in the portal phase was relatively reduced (Fig. 3b, Supplementary Fig. 4c).

### Anatomical morphology of cynomolgus monkey liver tumors with metastasis

**Fig. 4:**
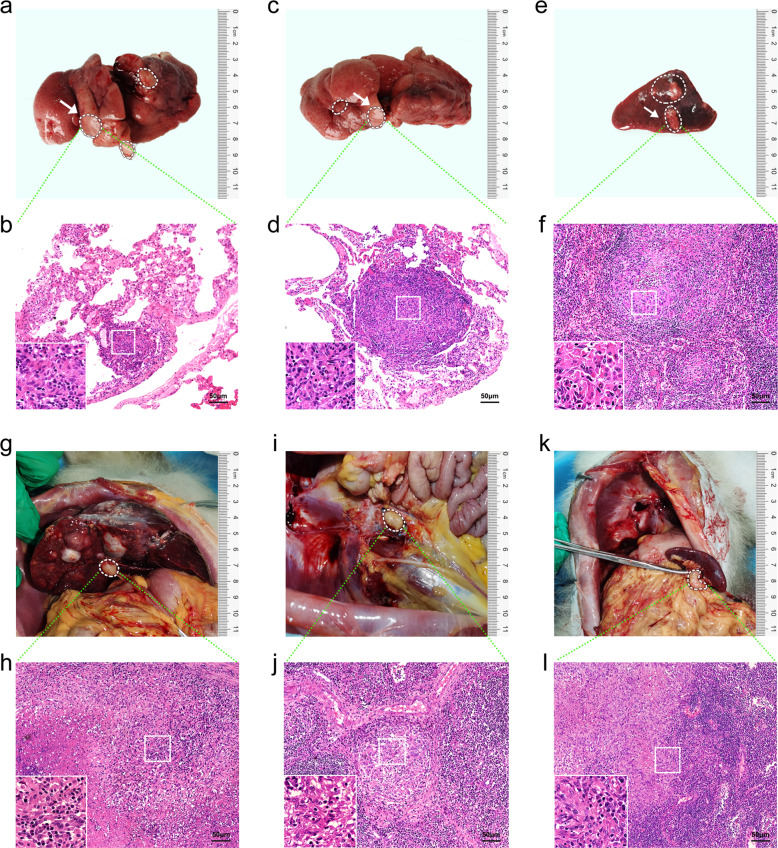
Modeling multiple metastatic tumors of primary liver cancer in cynomolgus monkeys using the CRISPR/Cas9 system. The cynomolgus monkeys were anesthetized and euthanized, and the distant organs with tumor metastasis were removed for HE and IHC staining when the liver tumor sizes were >2.0 cm in diameter on the ultrasound. **a** Left lung metastases were visible, and white circle indicated tumor lesions. **b** Pathological HE staining confirmed metastatic hepatocellular carcinoma. **c** Right lung metastases were visible, and white circle indicated tumor lesions. **d** Pathological HE staining confirmed metastatic hepatocellular carcinoma. **e** Splenic lymph node metastases were visible, and white circle indicated tumor lesions. **f** Pathological HE staining confirmed metastatic hepatocellular carcinoma. **g** Hilar lymph node metastasis was visible, and white circle indicated tumor lesions. **h** Pathological HE staining confirmed metastatic hepatocellular carcinoma. **i** Abdominal para-aortic lymph node metastasis was visible, and white circle indicated tumor lesions. **j** Pathological HE staining showed metastasis of liver cancer in the lymph nodes of the abdominal aorta. **k** Splenic lymph node metastasis was visible, and white circle indicated tumor lesions. **l** Pathological HE staining showed splenic lymph nodes of liver cancer metastasis. The insets were high-magnification views (×1000)

The cynomolgus monkeys were executed by euthanasia after the tumor sizes were >2.0 cm in diameter on the ultrasound. There were many spherical hard nodules of varying sizes 0.5–3.5 cm in diameter on the surface of the liver, scattered and distributed, with incomplete capsules, unclear boundaries, and gray–white cut surfaces (Fig. 3c, Supplementary Fig. 4a, i). There were multiple scattered hard nodules in the lungs and spleen with a diameter from 0.5 to 1.0 cm (Fig. 4a, c, e). Hilar lymph nodes, splenic lymph nodes, and para-aortic lymph nodes were enlarged and hard (Fig. 4g, i, k).

### Histopathology of cynomolgus monkey liver tumors with metastasis

We subsequently checked the pathology of liver tumors induced by the CRISPR/Cas9- delivery. Hematoxylin and eosin (HE) staining of liver sections from the cynomolgus monkeys were performed. As shown in the representative images from the CRISPR/Cas9-injected cynomolgus monkey, the histopathological features for tumor formation were noted under an optical microscope. The liver lobules had no certain structure, which was disordered, and the cells were arranged loosely, obviously irregular, and different in size. The cytoplasms of liver cells were loose and empty, and transparent, with large and irregular nuclei, and the ratio of nucleoplasm was significantly increased. The chromatin was obviously thickened, the HE staining was deepened (Fig. 3d, Supplementary Fig. 4b, e–h, j). In the lung metastases, the cells were arranged disorderly, with large morphological variations, more spindle cells, larger nuclei, cell abnormalities, and increased pathological mitotic phases. The cytoplasms were small, the nucleoli were large and irregular (Fig. 4b, d). The tissue structure of the spleen and lymph nodes was destroyed, and the atypical cells in clusters, nests, or sheets appeared (Fig. 4f, h, j, l).

### Phenotype alteration in cynomolgus monkey liver tumor tissues

**Fig. 5:**
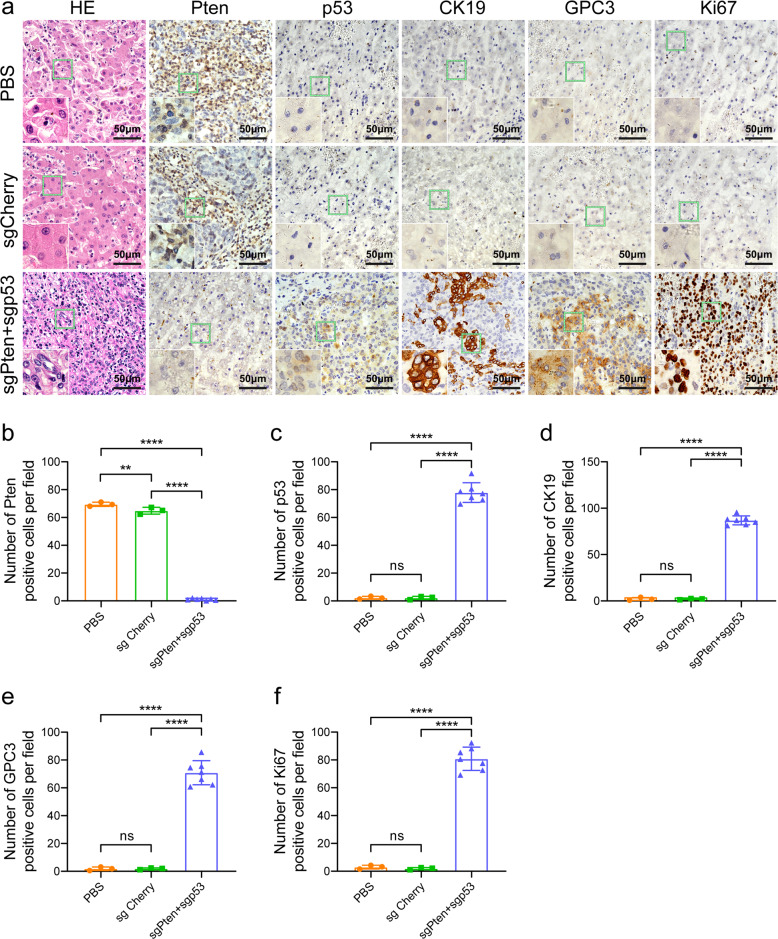
Representative images of HE and IHC staining of liver cancer from cynomolgus monkeys post-CRISPR/Cas9 delivery. The cynomolgus monkeys were anesthetized and euthanized, and the livers were removed for HE and IHC staining when the tumor sizes were >2.0 cm in diameter on the ultrasound. **a** HE and IHC staining of liver sections for examination of Pten, p53, GPC3, CK19, and Ki67 expression in representative cynomolgus monkeys. **b**–**f** The quantification of the percentage of Pten, p53, GPC3, CK19, and Ki67-positive cells in PBS, sgCherry, and sgPten+sgp53 indel groups, respectively. ^ns^*P* > 0.05, **P < 0.01, ****P < 0.0001. The error bars represent SD. The insets were high-magnification views (×1000)

**Fig. 6:**
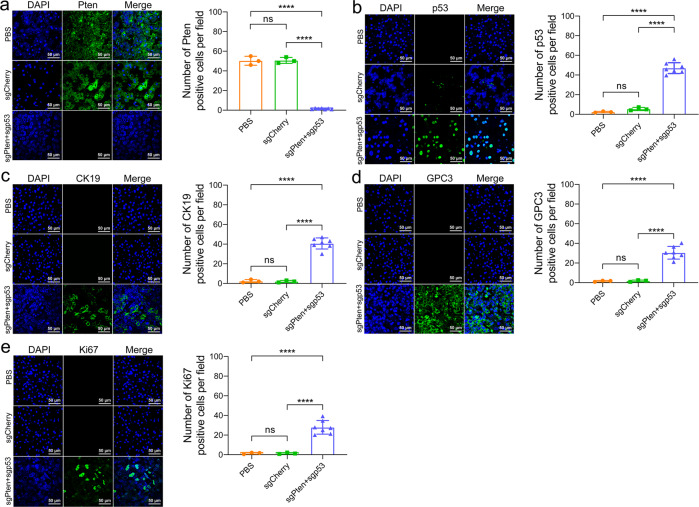
Representative images of IF staining of liver cancer from cynomolgus monkeys post-CRISPR/Cas9 delivery. The cynomolgus monkeys were anesthetized and euthanized, and the livers were removed for IF staining when the tumor sizes were >2.0 cm in diameter on the ultrasound. **a**–**e** IF staining of liver sections for examination of Pten, p53, GPC3, CK19, and Ki67 expression in representative cynomolgus monkeys. The quantification of the percentage of Pten, p53, GPC3, CK19, and Ki67-positive cells in PBS, sgCherry, and sgPten+sgp53 indel groups, respectively. ^ns^*P* > 0.05, ****P < 0.0001. The error bars represent SD. The insets were high-magnification views (×600)

We further investigated the phenotypic alteration of liver tumors induced by the CRISPR/Cas9 delivery. Immunohistochemistry (IHC) and immunofluorescence (IF) staining for Pten, p53, cytokeratin 19 (CK19), glypican-3 (GPC3), and proliferation-related ki67 antigen (Ki67) of the liver sections from the same cynomolgus monkey were also conducted and observed under an optical and laser scanning confocal microscope, respectively. The results showed that the numbers of hepatocytes and bile duct epithelial cells negative for the Pten, positive for the p53 and Ki67 in the sgPten+sgp53 indel cynomolgus monkeys were significantly greater than those observed in the sgCherry or PBS-treated cynomolgus monkeys (Figs. 5, 6). Meanwhile, IHC and IF staining for GPC3 and CK19, two common liver tumor markers, displayed stronger signals in the sgPten+sgp53 indel cynomolgus monkeys in contrast to the sgCherry or PBS-treated cynomolgus monkeys (Figs. 5, 6).

### Serum tumor markers in cynomolgus monkeys with primary and metastatic liver cancers

**Fig. 7:**
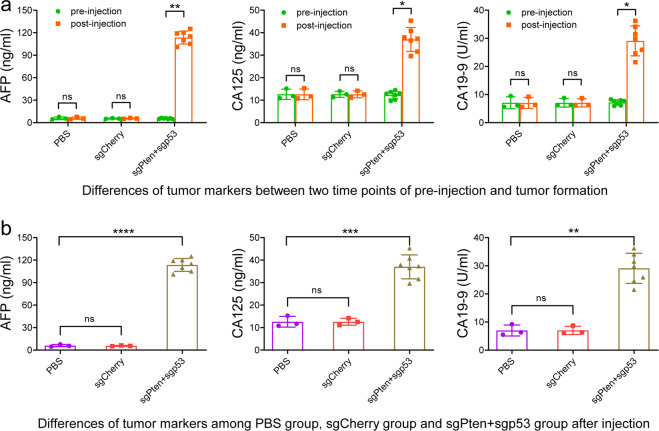
Serum levels of tumor markers in cynomolgus monkeys. Blood samples were drawn from the experimental cynomolgus monkeys before injection of the CRISPR/Cas9 system and when the tumor sizes were >2.0 cm in diameter on the ultrasound. Serum levels of three widely used tumor markers, AFP, CA125, and CA19-9, were measured by electrochemiluminescence immunoassay on a Roche E601 automatic analyzer. **a** Comparison of serum levels of AFP, CA125, CA19-9 of the sgPen+sgp53 group, sgCherry group, and PBS group between pre-injection of the CRISPR/Cas9 system and when the tumor sizes were >2.0 cm in diameter on the ultrasound. ^ns^*p* > 0.05, ***p < 0.001. **b** Differences in serum levels of AFP CA125, CA19-9 in the sgPen+sgp53 group, sgCherry group, and PBS group when the tumor sizes were >2.0 cm in diameter on the ultrasound. ^ns^*P* > 0.05, **P < 0.01, ***P < 0.001, ****P < 0.0001. The error bars represent SD

We assessed the serum levels of several tumor markers, AFP, CA125, and CA19-9. These proteins have been widely accepted as molecular markers for a broad range of human cancers, including liver cancers. The results indicated that the serum level of AFP was significantly increased in the sgPten+sgp53 indel cynomolgus monkeys, whereas no significant difference in AFP was observed pre- and post-injections of the CRISPR/Cas9 system in the sgCherry or PBS-treated group (Fig. 7a). Notably, the cynomolgus monkeys in the sgPten+sgp53 indel group post-CRISPR/Cas9 injection showed significantly elevated serum levels of CA125 and CA19-9 compared with pre-CRISPR/Cas9 injection (Fig. 7a). A comparative study of the tumor markers between the sgPten+sgp53 indel cynomolgus monkeys and the sgCherry or PBS group following the CRISPR/Cas9 injection revealed that serum levels of AFP, CA125, and CA19-9 were significantly increased in the sgPten+sgp53 indel monkeys compared with the sgCherry or PBS group. However, no significant difference was observed in serum levels of AFP, CA125, and CA19-9 after the CRISPR/Cas9 injection between the sgCherry or PBS group (Fig. 7b).

### WGS for off-target analysis

To monitor the risk of off-target mutations, we performed WGS on genomic DNA isolated from the PBMC (peripheral blood mononuclear cell) of the indel cynomolgus monkeys studied. WGS indicated that no mutations were revealed (Supplementary Fig. 3), suggesting high specificity of the CRISPR/Cas9 system for *Pten* and *p53*.

## Discussion

NHPs are recognized as more relevant animal models compared with rodents for cancer research and efficacy and safety assessment of anti-tumor drugs. The conventional methods for the generation of animal models with loss-of-function or gain-of-function mutations in cancer-related genes induced by targeting embryonic stem cells or transgenesis have been very challenging, tedious, and expensive in NHPs. This is mainly due to their low reproduction rate and long sexual maturation time. In this study, we first describe the potential of a new approach to quickly generate effective loss-of-function mutations in the *Pten* and *p53* genes in the livers of adult cynomolgus monkeys. We used the CRISPR/Cas9 genomic editing system, which was delivered by intrahepatic portal vein injection under ultrasound guidance. The major novel findings of this study are summarized as follows: (1) intrahepatic portal vein injection of the CRISPR/Cas9 components under the guidance of color ultrasound successfully delivered the genomic editing system for *Pten* and *p53* into the livers of cynomolgus monkeys; (2) the delivery of the CRISPR/Cas9 system into the livers led to mutations, including both deletions and insertions, in the *Pten* and *p53* genes of the cynomolgus monkey as confirmed by WGS; (3) the CRISPR/Cas9-mediated indels occurred at a high frequency, and were very easy to generate loss-of-function effects on the *Pten* and *p53* genes and primary and metastatic hepatoma formation as confirmed by anatomical morphology and histopathology; and (4) intrahepatic portal vein injection of the CRISPR/Cas9 system for Pten and p53 into the liver holds promise as an effective and feasible method for generation of an NHP model for liver cancer.

The CRISPR/Cas9 system is a novel genomic editing tool that has been successfully applied to the targeting of specific genes for knock-out, knock-in, or disruptions in a wide range of mammalian cell lines^22–27^ and embryonic cells of mice,^19^ rats,^19,28^ zebrafish,^17^ fruit flies,^29^ elegans,^30^ and cynomolgus monkeys.^31,32^ Compared with the delivery of a plasmid expressing the Cas9-sgRNA complex through the cell membrane, the physical delivery of the CRISPR/Cas9 system for in vivo application remains challenging. In vivo transfection is difficult to implement because the Cas9 fragment is longer and difficult to import. In addition, the body environment is complex and the Cas9 fragment is easily dispersed when administered by systemic intravenous injection and thus targeting is not always specific. Recently, it was reported that hydrodynamic injection of the CRISRPR/Cas9 system through the mouse tail vein can accommodate the larger size of plasmid DNA. This method led to successful CRISPR/Cas9-mediated insertion and deletion mutations of two tumor suppressor genes, *Pten* and *p53*, which resulted in liver tumor formation.^25^ However, further studies are required to fully evaluate the side effects of the CRISPR system in mice and other organisms.^25^ In an effort to efficiently and broadly enable the application of the CRISPR/Cas9 system in vivo, a Cas9 knock-in mouse model was created and designed to overcome challenges in the delivery of Cas9.^2^ They combined multiple sgRNAs specific for the top three mutated genes in lung adenocarcinoma, Kras, p53, and LKB1. The combination resulted in the development of lung adenocarcinomas in mice induced by loss-of-function multi-genetic cancer mutations in LKB1 and p53 and HDR-mediated Kras^G12D^ mutations.^2^ To date, the efficiency and safety of these delivery methods for the CRISPR/Cas9 system in NHPs remain to be determined. Notably, in our study, the CRISPR/Cas9-expressing adenoviruses were directly delivered to the livers of cynomolgus monkeys to achieve in situ gene editing through the intrahepatic portal vein under ultrasound guidance, which significantly increased more efficiency as well as higher specificity in vivo. This was because the CRISPR/Cas9 system was delivered directly to the liver, systemic dilution was eliminated and specificity of the desired genomic editing was greatly enhanced. Meanwhile, ultrasound can provide real-time dynamic observation of the injection process and the ultrasound-guided portal injection is controllable and highly precise. This delivery technology appears to have great potential to successfully pursue the desired target genes, thereby reducing possible side effects associated with hitting non-specific off-target genes. Furthermore, this technology could bypass the need for engineering embryonic cells and for breeding multiple mutant experimental animals to establish a more relevant animal model. Therefore, the ultrasound-guided delivery of the CRISPR system could represent a more efficient and safer strategy for in situ genomic editing of desired genes in the cynomolgus monkeys and other NHPs.

The two ways of tumor metastasis are blood circulation and lymphatic drainage. Extrahepatic metastasis is due to cancer cells involving hepatic veins and lymphatic vessels, then entering the systemic circulation and/or infiltrating and planting to various parts of the body, such as the lung, peritoneum, and lymph nodes. Among them, the lung is the most common. The lungs, spleen, and lymph nodes of monkeys with primary liver cancer we created had multiple scattered circular parenchymal lesions. This model should greatly speed up the discovery of new anti-cancer drugs and save research and development costs.

In the present study, the well-known tumor suppressor genes *Pten* and *p53* were chosen as the targets for disruption using the CRISPR/Cas9 genomic editing system in the cynomolgus monkey. Our data demonstrated that delivery of Adeno-Pten-sgRNA and Adeno-p53-sgRNA into the livers of adult cynomolgus monkeys by intrahepatic portal vein injection-induced high mutations in *Pten* and *p53* and successfully gave rise to primary and metastatic hepatoma. This appeared to be very successful to produce phenotype alterations of liver tumors. Targeting *Pten* and *p53* resulted in tumor formation identified by histopathological HE staining, no expression of Pten, and overexpression of mutant p53 (It should be noted that the physical and chemical properties of mutant p53 are different from wild-type p53.^33^ Normally, the mutant p53 has higher products in proliferating cells, better stability and longer half-life (1.4–7 h), which can be detected by IHC. However, the wild-type p53 product in the cell is very low, extremely unstable, and has a short half-life of only a few minutes, so it cannot be detected at all), as well as GPC3, CK19, and Ki67 in liver tumor tissues, and significantly elevated serum levels of tumor markers, including AFP, CA125, and CA19-9. Ki67 is present in active cell cycle phases but absent in the G0 phase and is well-accepted as a cellular proliferation marker.^24,34–37^ Thus, the presence of high levels of Ki67 observed following the CRISPR-mediated disruption of the *Pten* and *p53* genes in the liver of the mutant cynomolgus monkey was not surprising. As two common liver tumor markers,^38–40^ GPC3 and CK19 were over-expressed in liver tumor tissues obtained from *Pten* and *p53* mutant cynomolgus monkeys. The serum tumor markers, AFP,^41–44^ CA125, and CA19-9,^45–48^ were chosen because these proteins have been identified in various human cancers, including liver cancer. These proteins were detected in each of seven *Pten* and *p53* mutant monkeys. These findings suggested that primary and metastatic tumors were induced in the livers by 7–10 months following the delivery of the CRISPR system.

Although the CRISPR/Cas9 genomic editing system can cleave genomic DNA at directed target sites, concerns remain regarding unwanted mutations associated with off-target cleavage in vivo. In this study, WGS showed no mutations were detected, indicating a high specificity of the CRISPR/Cas9 system for targeting Pten and p53 in these experimental monkeys. Similar results were also observed in monkey embryo cells.^31^

A potential limitation of this study is that the mechanism by which CRISPR induces liver cancer metastasis is unclear. This may need to be further elucidated from the perspective of immunology and molecular biology in future studies. Additionally, other potential off-target events might exist following as yet unidentified processes. Therefore, it is necessary to add more samples to continue off-target analysis of WGS in the future. Furthermore, the CRISPR/Cas9 genomic editing technique is an emerging genomic tool and, therefore, is still in its infancy. Long-term safety remains unclear and will need to be evaluated in various NHPs. Although no adverse effects on the digestive system were observed in our study, the long-term toxicity, and impact of genetic interruption and off-targeting on the next generation will need to be assessed in cynomolgus monkeys and other NHPs.

In summary, we demonstrated that *Pten* and *p53* gene mutations could be efficiently produced in the liver of cynomolgus monkeys by intrahepatic portal vein injection of the CRISPR/Cas9 genomic editing system. Moreover, this novel approach overcomes the challenges of traditional methods for generating an animal model with genetic mutations and bypasses the requirement for breeding multiple mutant animals.

Taken together, our results underscore that in situ adult monkey gene editing is a powerful and feasible tool that can effectively disrupt other desired tumor suppressor genes or oncogenes, and quickly establish primary and metastatic tumors in NHPs. This novel approach has the advantages of celerity, high-efficiency, safety, targeting, and so on. In addition, the technology can be used not only for the establishment of fatty liver, cirrhosis, hepatitis, and other liver disease models, but also for the delivery of CRISPR/Cas9 system into other organs (such as lung, kidney, etc) under the ultrasound guidance through the relevant vessels to create corresponding disease models, which are applied to study new gene diagnosis and therapeutic strategy like drug discovery. Therefore, the primate disease models using in situ adult monkey gene editing are the most suitable screening platform for precise diagnosis and therapeutic strategy.

## Materials and methods

### Institutional approval

In this study, the cynomolgus monkey facility was notarized by the Association for Assessment and Accreditation of Laboratory Animal Care (AAALAC) International and all experimental procedures and animal maintenance were permitted by the Institutional Animal Care and Use Committee of National Center for International Research of Biological Targeting Diagnosis and Therapy, Guangxi Medical University, Nanning, Guangxi, China.

### Chemicals and reagents

DMEM, Opti-MEM, fetal bovine serum (FBS), and the penicillin–streptomycin solution were purchased from GIBCO/Thermo Fisher Scientific (Waltham, MA, USA). The anti-Pten, anti-p53, anti-GPC3, anti-CK19, and anti-Ki67 antibodies were from Abcam (Abcam, Cambridge, UK). T7 ligase, Q5 high-fidelity DNA polymerase, and T7E1 were purchased from New England Biolabs (Ipswich, MA, USA) and the Plasmid Extraction Kit and Genome DNA Isolation Kit were from Omega (Washington DC, USA). The PCR Purification Kit was from Axygen (Union City, CA, USA) and the SuperCore^TM^ Biopsy Needle MCXS1815LX was obtained from Argon Medical Inc. (Washington DC, USA). The percutaneous ethanol injection therapy (PEIT) needle was purchased from Hakko Co., Ltd (Nagano, Japan) and the Etamsylate Injection (2 mL: 0.5 g) solution was from Huazhong Pharmaceutical Co., Ltd (Wuhan, Hubei, China). The Shu Mianning II Injection solution was obtained from Nanjing Agricultural University (Nanjing, Jiangsu, China). Alpha Fetal Protein Assay Kit, Cancer Antigen 125 Assay Kit, and Cancer Antigen 19-9 Assay Kit were purchased from Roche Diagnostics GmbH (Mannheim, Germany).

### Experimental animals

Healthy male cynomolgus monkeys, ranging in age from 4.5 to 6 years and weighing from 3.2 to 6.0 kg, were purchased from the Primate Center for Medical Science, Changchun Biotech (Fangchenggang, Guangxi, China). This facility is accredited by AAALAC International. Eight cynomolgus monkeys were used for the *Pten* and *p53* gene editing (sgPten + sgp53 group), three for Cherry gene editing (sgCherry group), and three for PBS as a control group (PBS group). All study protocols involving experimental primates were reviewed and approved by the Animal Care & Ethics Committee of Guangxi Medical University.

### Cell line and culture

The COS-7 cell line, derived from kidney tissue of the African green monkey, was purchased from the Cell Bank of Chinese Academy of Sciences (Shanghai, China). The COS-7 cells were cultured in DMEM supplemented with 10% (v/v) FBS, 100 U/mL penicillin, and 100 μg/mL streptomycin. All cells were maintained in an incubator under an atmosphere of 5% CO_2_ at 37 °C.

### Construction of CRISPR/Cas9-expressing adenovirus

The plasmid “pAdeno-CMV-mCherry-T2A-3Flag-hCas9” (Obio Technology, Shanghai, China), ALB promoter replaced the mCherry and T2A sequence, and Cas9 genes were driven by ALB promoter. A 3xFlag tag was fused to the N terminus of the codon humanized Cas9 gene. Based on “pAdeno-U6-CCDB- CMV-EGFP” vector, the adenovirus shuttle plasmid “pAdeno-ALB-Pten gRNA1-ALB-Pten gRNA2-Pten donor” was constructed via two steps: First, ALB promoter, sgRNA sequence targeting *Pten* were synthesized and inserted between two BsmBI sites to obtain plasmid “pAdeno-ALB-Pten sgRNA-CMV- EGFP”. Then, a donor sequence with *Pten* mutations (G129E) was synthesized and inserted between BamHI and HindIII sites to replace the segment of CMV-EGFP. The adenovirus shuttle plasmid “pAdeno-ALB-p53 gRNA-p53 Donor” was constructed on the basis of “pAdeno-U6-CCDB-CMV-EGFP” vector via two steps: first, ALB promoter and gRNA sequence targeting *p53* were synthesized and inserted between two BsmBI sites. Second, a donor sequence with *p53* mutations (R248G, R249G) was synthesized and inserted between BamHI and HindIII sites to replace the segment of CMV-EGFP. Above adenovirus shuttle plasmids with the gene of interest were co-transfected into HEK-293 cell with helper plasmid pBHGlox (delta) E1, 3Cre to produce recombinant adenovirus particles according to the standard protocol of AdMax System (Microbix Biosystems Inc, Toronto, Canada).

### Transfection of the CRISPR/Cas9 system

COS-7 cells were seeded in six-well plates at a cell density of 1.0 × 10^6^ cells/well 1 day prior to transfection and cultured to 70% confluences. COS-7 cells were transfected with 0.5 MOI Adeno-Pten-sgRNA with Adeno-Cas9, Adeno-p53-sgRNA with Adeno-Cas9, and/or Adeno-Cherry-sgRNA with Adeno-Cas9. At 48 h post transfection, COS-7 cells were harvested and genomic DNA was extracted for subsequent sequencing.^25^

### T7E1 digestion and TA cloning for sequencing

PCR amplification of the *Pten* target sites was performed in a reaction containing COS-7 genomic DNA as the template, a pair of Pten-F and Pten-R primers, and Q5 high-fidelity DNA polymerase. The PCR cycling profile was as follows: 98 °C, 30 s; 35 cycles (98 °C, 10 s; 57 °C, 15 s; 72 °C, 20 s); 72 °C, 2 min; 95 °C, 5 min; slowly re-annealing using the following temperature program: temperature was reduced to 85 °C (2 °C decrease/s); temperature was further reduced more slowly to 25 °C (0.1 °C decrease/s). PCR amplification of the *p53* target sites was performed in a reaction containing COS-7 genomic DNA as the template, a pair of p53-F and p53-R primers, and Q5 high-fidelity DNA polymerase. The PCR cycling profile was as follows: 98 °C, 30 s; 35 cycles (98 °C, 10 s; 60 °C, 15 s; 72 °C, 20 s); 72 °C, 2 min; 95 °C, 5 min; slowly re-annealing using the following temperature program: the temperature was reduced to 85 °C (2 °C decrease/s); the temperature was further reduced more slowly to 25 °C (0.1 °C decrease/s). The PCR products were subjected to the T7E1 assay. In brief, 10 μL of the PCR products were immediately digested with 0.25 μL of T7E1 for 30 min at 37 °C, followed by electrophoresis analysis in a 2% DNA gel. To further verify the mutation of the *Pten* and *p53* genes, the purified PCR products were cloned into the T-vector and subsequently transformed into competent cells, after which 30 single colonies were randomly picked for sequencing.

### Delivery of the CRISPR/Cas9 system into the liver through intrahepatic portal vein injection guided by color ultrasound

The sgPten + sgp53 group consisted of eight cynomolgus monkeys (*n* = 8), each monkey was injected Adeno-Pten-sgRNA (1 × 10^11^ pfu/kg), Adeno-p53-sgRNA (1 × 10^11^ pfu/kg) and Adeno-Cas9 (2 × 10^11^ pfu/kg). The sgCherry group consisted of three cynomolgus monkeys (*n* = 3), each monkey was injected Adeno-Cherry-sgRNA (1 × 10^11^ pfu/kg) and Adeno-Cas9 (1 × 10^11^ pfu/kg). PBS group consisted of three cynomolgus monkeys (*n* = 3), each monkey was injected with equal PBS. The CRISPR/Cas9 system was delivered into the liver of cynomolgus monkeys through the intrahepatic portal vein under the guidance of a portable ultrasonic device (GE Logic E9, USA). In brief, the cynomolgus monkeys were anesthetized with intramuscular injection of Shu Mianning II (0.1 mL/kg) and Etamsylate (8.75 mg/kg). Following the successful anesthesia, each was fixed in a supine position on an operating table. Under ultrasonic guidance, the sagittal section of the left intrahepatic portal vein was pinpointed, and PEIT needle (21 G × 200 nm) was inserted into the venous lumen. After the needlepoint in the lumen was confirmed by a bit of blood drawn, Adeno-Pten-sgRNA and Adeno-p53-sgRNA dissolved in 400 μL of normal saline (0.9% NaCl) were manually injected into each liver of the six experimental cynomolgus monkeys or the same amount of Adeno-Cherry-sgRNA into each liver of the control monkeys. An additional 1 mL of normal saline was subsequently injected into the liver of each cynomolgus monkey. The successful delivery of the CRISPR/Cas9 system through intrahepatic portal vein injection can be indicated by a strong echo signal captured by B ultrasound. Upon completion, PEIT needle was withdrawn from the body under real-time ultrasound guidance. After all, operations were completed, ultrasonography was used to examine the liver and surrounding organs for detection of complications such as bleeding or organ damage.

### Contrast-enhanced ultrasound

The probe was fixed on the liver section where the lesion was located, and two-dimensional ultrasound was routinely performed, and 0.5 mL/kg Sono Vue contrast by Bracco Suisse SA was injected through the vein of the lower limb.

### Enhanced CT scan

GE CT750HD CT machine was used for upper abdominal CT plain scan and multi-phase dynamic enhanced scan; 0.5 mL/kg contrast agent (60% meglumine diatrizoate) was injected through lower extremity vein at a flow rate of 0.5 mL/s, and then enhanced scan was performed. Arterial phase and venous phase scanning were started at 18 s and 48 s after injection of contrast agent. Parameters: tube voltage 120 kV, tube current 150 MV, slice spacing 1.25 mm, slice thickness 1.25 mm.

### Ultrasound-guided liver biopsies

Under sedation achieved by intramuscular injection of Shu Mianning II (0.1 mL/kg) and Etamsylate (8.75 mg/kg), a percutaneous liver biopsy was performed when the tumor masses were found on ultrasound. A biopsy needle was guided by B ultrasound to obtain two hepatic biopsies with each ~1.5 cm in length from the left lobe and right lobe of the liver of each cynomolgus monkey. During the procedure, the depth and position of the needle were adjusted as needed, according to the virtual B ultrasound scan. The biopsy samples were immediately frozen in liquid nitrogen for genomic DNA isolation.

### WGS for analysis of indels and off-target

Genomic DNA was isolated from the liver tumor tissues obtained when the tumor masses were found on ultrasound and used for WGS, which was performed on an Illumina MiSeq System according to the instructions provided by Illumina (San Diego, CA, USA) using the 2 × 300 bp paired-end method, in which 2 × 300 bp refers to the length of both ends of the fragment as 300 bp. The Illumina MiSeq Control Software (MCS; San Diego, CA, USA) was used for WGS reads. The resulting data were imported and analyzed by Cutadap, Pandaseq, Bwa, and Samtools software of GENEWIZ (Suzhou, Jiangsu, China) for *Pten* and *p53* insertions and deletions mediated by the CRISPR/Cas9 system. Potential off-target sequences of *Pten* and *p53* sgRNAs were identified using the CRISPR design tool^49^ (http://www.rgenome.net/cas- offinder/), and the BLAST tool^50^ (http://www.ncbi.nlm.nih.gov/genome/10731) against the 23 bp sequence (sgRNA + PAM). COS-7 cells were transiently transfected with Adeno-Pten-sgRNA with Adeno-Cas9 and Adeno-p53-sgRNA with Adeno-Cas9 for 48 hr and total genomic DNA was extracted and the *Pten* and *p53* loci were analyzed by the T7E1 assay.

### Histopathology, IHC, and IF

When the diameter of the tumor was ~2.0 cm, the cynomolgus monkey was euthanized, the liver tumors were dissected out and fixed in 4% paraformaldehyde overnight and embedded in paraffin wax and then were sectioned at 3 μm thickness. HE staining of the liver sections was performed for histopathology. For measurement of selected tumor markers, the slides were incubated with an anti-Pten primary antibody (Abcam, Cambridge, UK; 1:100 dilution), and anti-p53 primary antibody (Abcam, Cambridge, UK; 1:50 dilution), an anti-GPC3 primary antibody (Abcam, Cambridge, UK; 1:500 dilution), an anti-CK19 primary antibody (Abcam, Cambridge, UK; 1:200 dilution), or an anti-Ki67 antibody (Abcam, Cambridge, UK; 1:250 dilution) at 37 °C for 1 hr. IHC detection of the levels of Pten, p53, GPC3, CK19, and Ki67 protein expression was performed using laser scanning confocal microscopy (Nikon, Japan). For measurement of IF for selected tumor markers, the slides were incubated with an anti-Pten primary antibody (Abcam, Cambridge, UK; 1:100 dilution), an anti-p53 primary antibody (Abcam, Cambridge, UK; 1:50 dilution), an anti-GPC3 primary antibody (Abcam, Cambridge, UK; 1:500 dilution), an anti-CK19 primary antibody (Abcam, Cambridge, UK; 1:200 dilution), or an anti-Ki67 antibody (Abcam, Cambridge, UK; 1:250 dilution) overnight at 4 °C. Slides were washed and incubated in fluorescently conjugated secondary antibodies (Abcam, Cambridge, UK; 1:350 dilution) at 37 °C for 30 min. Cell nuclei were counterstained with DAPI at room temperature for 3 min. IF detection of Pten, p53, GPC3, CK19, and Ki67 protein expression was performed using laser scanning confocal microscopy (Nikon, Japan).

### Electrochemiluminescence

Blood samples (4 mL) were collected from the femoral veins of cynomolgus monkeys before injection of the CRISPR/Cas9 system and when the diameter of the tumor was ~2.0 cm. Serum samples were isolated by centrifugation at 3000 rpm for 10 min and then collected in 2 mL EP tubes. Serum levels of selected tumor markers, including AFP, CA125, and CA19-9, were determined by using a Roche E601 Electrochemiluminescence Immunoassay Instrument (Roche. Mannheim, Germany) following the user manual.

### Statistical analysis

GraphPad Prism software v 5.0 (San Diego, CA, USA) was used for statistical analysis. Data are expressed as mean values ± SD. Comparisons between treatment and control groups were conducted using one-way ANOVA and a *p* value < 0.05 was considered statistically significant.

### Further materials and methods

For details, a supplementary section is provided in the supplementary information of this manuscript.

## Supplementary information

Supplementary Materials

## Data Availability

All data and methods in this study are available upon request.
