# Supplementary material for: Generation of in situ CRISPR-mediated primary and metastatic cancer from monkey liver
Source: Signal Transduct Target Ther. 2021 Dec 3;6:411. doi: 10.1038/s41392-021-00799-7 (PMC8640017; doi:10.1038/s41392-021-00799-7)
Supplement: Supplementary file 1 — Supplementary Materials [file 41392_2021_799_MOESM1_ESM.docx]

Supplementary Materials for

**Generation of *in situ* CRISPR-mediated primary and metastatic cancer from monkey liver**

Liping Zhong^1, †^, Yong Huang^1, †^, Jian He^1^, Nuo Yang^1^, Banghao Xu^2^, Yun Ma^3^, Junjie Liu^4^, Chao Tang^1^, Chengpiao Luo^3^, Pan Wu^1^, Zongqiang Lai^1^, Yu Huo^1^, Tao Lu^1^, Dongni Huang^1^, Wenlin Gong^1^, Lu Gan^1^, Yiqun Luo^1^, Zhikun Zhang^1^, Xiyu Liu^1^, Yongxiang Zhao^1,^*

**This PDF file includes:**

**Supplementary Figure S1-S4 with Legends**

**Supplementary Table S1-S2**

**Supplementary Video S1- S3**

**Supplementary Figure S1**

**
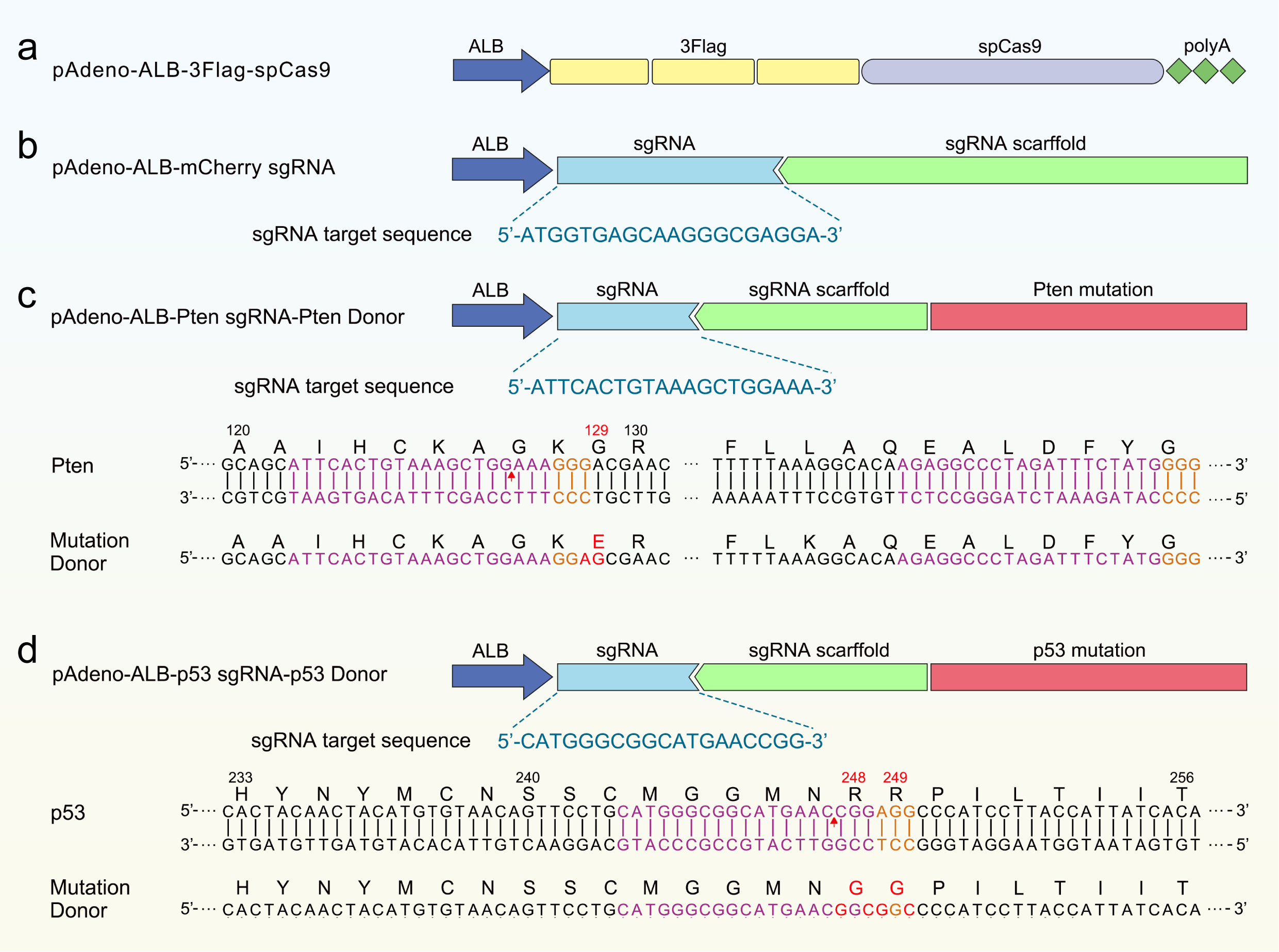
**

**Supplementary Figure 1. Schematic diagram of CRISPR/Cas9-expressing adenovirus. (a)** Schematic representation of the pAdeno-ALB-3Flag-spCas9 expressing Cas9. **(b)** Schematic representation of the pAdeno-ALB-mCherry-sgRNA expressing mCherry sgRNA. **(c)** Schematic representation of the pAdeno-Pten-sgRNA expressing *Pten* sgRNA. The vector was designed using 1 sgRNA with the N20NGG pattern to target mutation G129E. **(d)** Schematic representation of the pAdeno-p53-sgRNA expressing *p53* sgRNA. The vector was designed using the N20NGG pattern to simultaneously target mutation R248G and R249G.

**Supplementary Figure. S2**

**
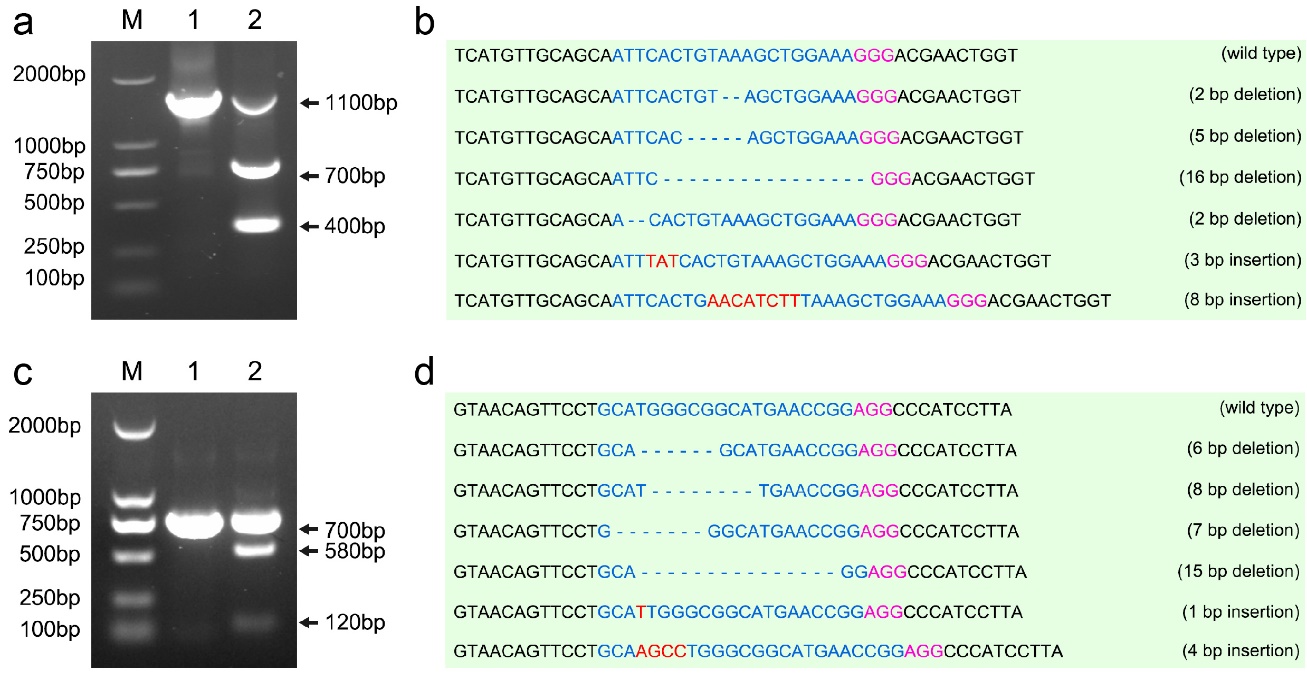
**

**Supplementary Figure 2. Gene disruption for *Pten* and *p53* using CRISPR/Cas9 system in COS-7 cell line**. COS-7 cells were transfected with 0.5 MOI Adeno-Pten-sgRNA with 0.5 MOI Adeno-Cas9, Adeno-p53-sgRNA with 0.5 MOI Adeno-Cas9 and / or Adeno-Cherry-sgRNA with 0.5 MOI Adeno-Cas9 following the manufacturer’s protocol. At 48 hr after transfection, cells were harvested and total genomic DNA was isolated for subsequent PCR application, T7E1 cleavage assay and sequencing of *pten*-targeting locus and the *p53*-targeting locus. **(a)** T7E1 cleavage assay of the edited *Pten* gene after transfection of COS-7 cells with Adeno-Pten-sgRNA. There were two cleavage bands in target gene, 580 bp and 120 bp, respectively. M: Marker; lane 1: before digestion with T7E1; lane 2: after T7E1 digestion. **(b)** Representative sequences of indels. Seven indels were deletions denoted by dotted blue lines and insertions indicated by red capital letters. **(c)** T7E1 cleavage assay of the edited *p53* gene after transfection of COS-7 cells with Adeno-p53-sgRNA. There were two cleavage bands in target gene, 1100 bp and 400 bp, respectively. M: Marker; lane 1: before digestion with T7E1; lane 2: after T7E1 digestion. **(d)** Representative sequences of indels. Seven indels were deletions denoted by dotted blue lines and insertions indicated by red capital letters.

**Supplementary Figure S3**


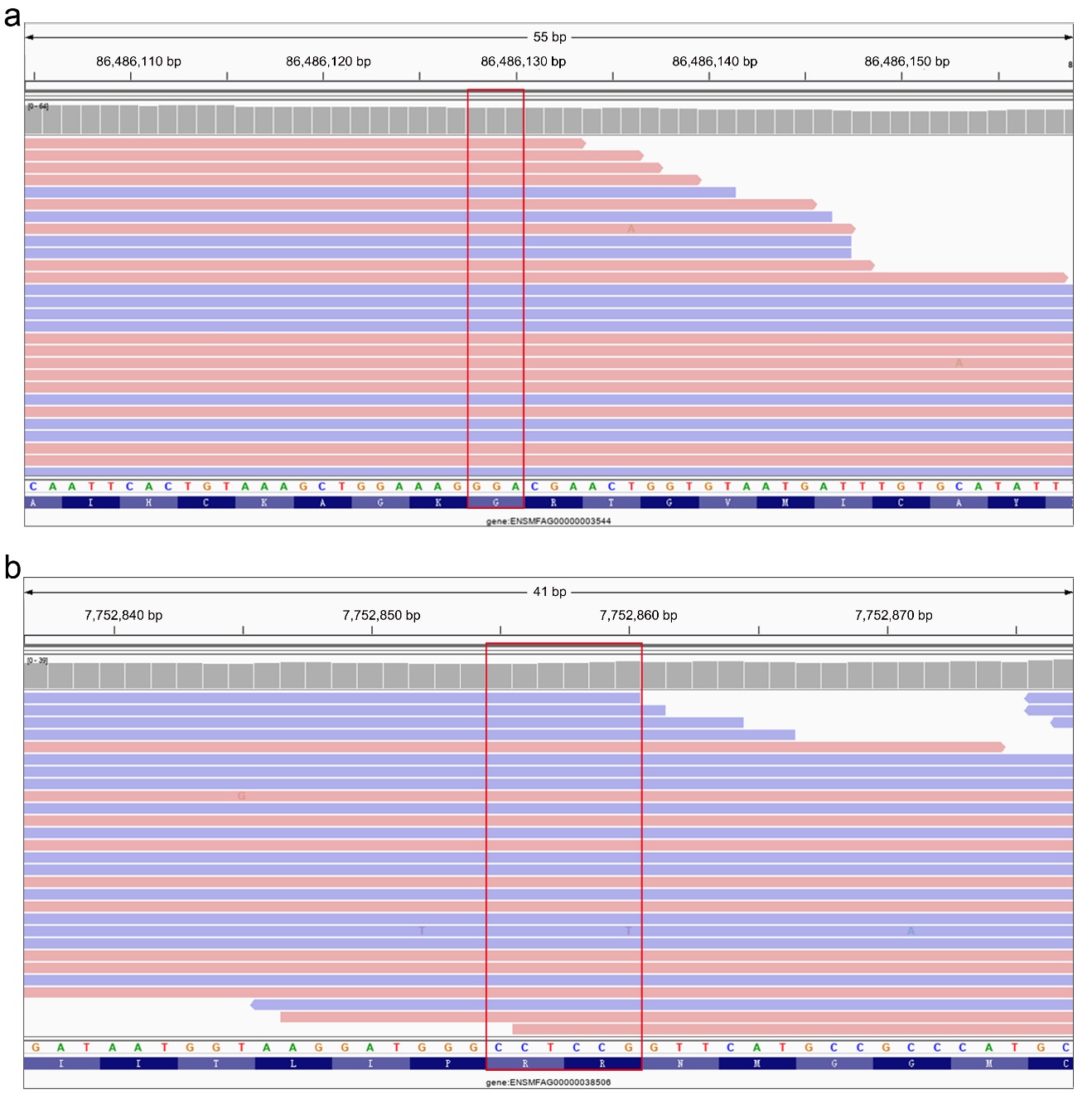


**Supplementary Figure 3. Whole-genome sequencing of the *Pten and p53* genes in PBMC of cynomolgus monkeys**. **(a)** WGS of the *Pten* gene in PBMC of cynomolgus monkeys revealed no off-target phenomenon was found. **(b)** WGS of the *p53* gene in PBMC of cynomolgus monkeys revealed no off-target phenomenon was found.

**Supplementary Figure S4**


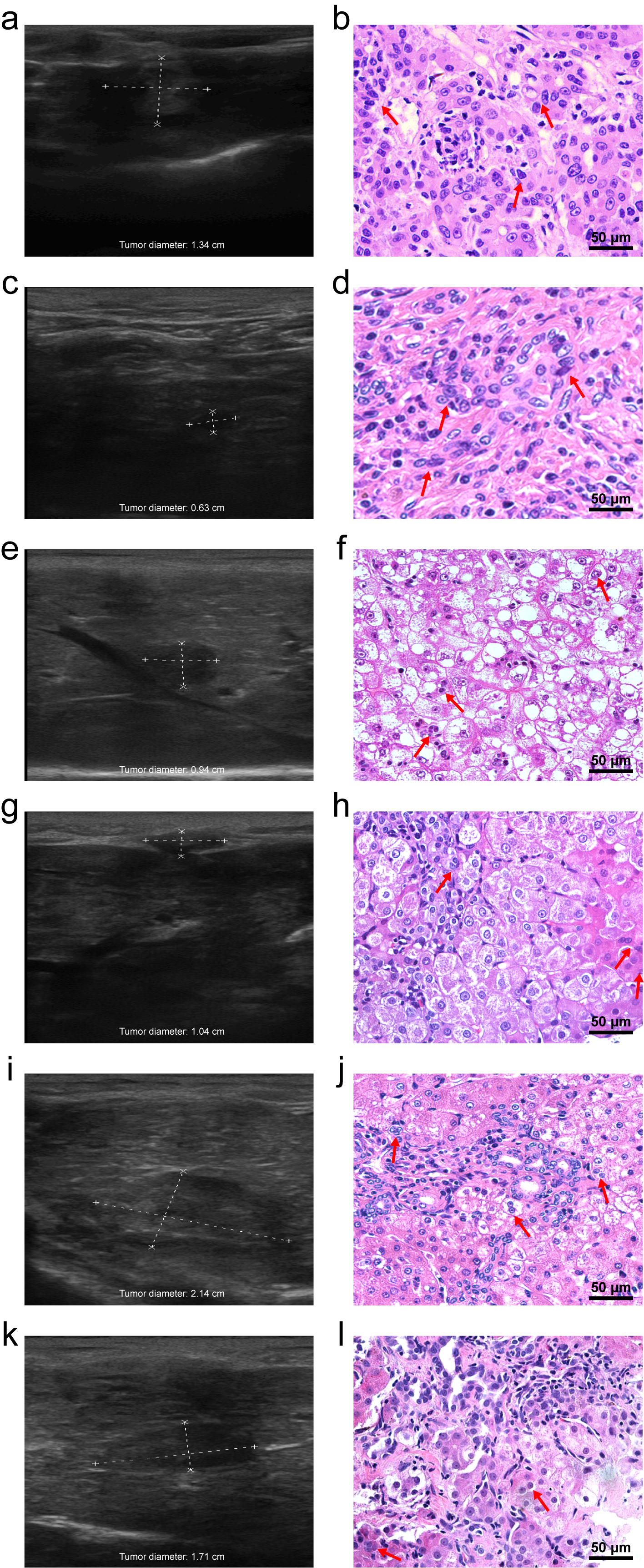


**Supplementary Figure 4. Ultrasound imaging and pathological examination of liver cancer in situ in cynomolgus monkeys.** **(a, c, e, g, h, i)** Representative of ultrasound images of liver cancer cynomolgus monkeys. **(b, d, f, h, j, l)** Pathological HE staining confirmed liver cancer in situ in cynomolgus monkeys, the red arrow points to the liver cancer cells. The insets were high-magnification views (×400).

**Table S1. Sequences of the gRNAs and primers used in this study**

| Name | Sequence (5’ to 3’) |
| --- | --- |
| p53 Sense (gRNA) | CATGGGCGGCATGAACCGG |
| p53-F (PCR p53 genomic region) | TACGATACAAGGCTGTTAGAGAG |
| p53-R (PCR p53 genomic region) | CTATTAATAACTAATGCATGGC |
| Pten Sense (gRNA) | ATTCACTGTAAAGCTGGAAA |
| Pten -F (PCR Pten genomic region) | TACGATACAAGGCTGTTAGAGAG |
| Pten -R (PCR Pten genomic region) | CTATTAATAACTAATGCATGGC |
| mCherry Sense (gRNA) | ATGGTGAGCAAGGGCGAGGA |
| mCherry Antisense (gRNA) | CTTGTACAGCTCGTCCAT |

**Table S2. Key Resources of Experiments**

| REAGENT or RESOURCE | | | SOURCE | IDENTIFIER | |
| --- | --- | --- | --- | --- | --- |
| Antibodies | | |  |  | |
| anti-Pten antibody | | | Abcam | ab170941 | |
| anti-p53 antibody | | | Abcam | ab32389 | |
| anti-GPC3 antibody | | | Abcam | ab207080 | |
| anti-CK19 antibody | | | Abcam | ab52625 | |
| anti-Ki67 antibody | | | Abcam | ab16667 | |
| Goat anti-Rabbit IgG Alexa Flour @ 488 | | | Abcam | ab150073 | |
| DAPI | | | Solarbio | C0065 | |
| Bacterial and Virus Strains | | |  |  | |
| pAdeno-U6-spgRNA v2.0-CMV-EGFP | | | Obio | H5066 | |
| Biological Samples | | |  |  | |
| Liver biopsy tissues of cynomolgus monkeys | | | The Primate Center for Medical Science, Changchun Biotech | Stock number: Q1305083 Q1404001 Q1408039 Q1401107 Q1310019 Q1412045 Q1408035 Q1111033 Q1006051 Q1207025 Q1112013 Q1207049  Q1202023  Q1201023 | |
| Blood samples from cynomolgus monkeys | | | The Primate Center for Medical Science, Changchun Biotech | Stock number: Q1305083 Q1404001 Q1408039 Q1401107 Q1310019 Q1412045 Q1408035 Q1111033 Q1006051 Q1207025 Q1112013 Q1207049  Q1202023  Q1201023 | |
| Chemicals, Peptides, and Recombinant Proteins | | | | | |
| T7 ligase | | | New England Biolabs | M0318S | |
| Q5 high-fidelity DNA polymerase | | | New England Biolabs | M0493S | |
| Etamsylate Injection (2 ml: 0.5 g) solution | | | Huazhong Pharmaceutical Co. Ltd | H42021494 | |
| T7 Endonuclease 1 | | | New England Biolabs | M0302L | |
| ShuMianning II Injection solution | | | Nanjing Agricultural University | N/A | |
| Critical Commercial Assays | | |  |  | |
| Plasmid Extraction Kit | | | Omega | D2500-02 | |
| Genome DNA Isolation Kit | | | Omega | D6908-00 | |
| PCR Purification Kit | | | Axygen | AxyPrep^TM^: 34014KBI | |
| Alpha Fetal Protein Assay Kit | | | Roche Diagnostics GmbH | YZB/GER 5307-2014 | |
| Cancer Antigen 125 Assay Kit | | | Roche Diagnostics GmbH | YZB/GER 1568-2015 | |
| Cancer Antigen 19-9 Assay Kit | | | Roche Diagnostics GmbH | YZB/GER 5395-2014 | |
| DNA Library Prep Kit | | | New England Biolabs | E7645S | |
| Experimental Models: Cell Lines | | | | | |
| COS-7 cell line | ATCC | | | | CRL-1651^TM^ |
| Experimental Models: Organisms/Strains | | | | | |
| Healthy male cynomolgus monkeys | | The Primate Center for Medical Science, Changchun Biotech | | Stock number: Q1305083 Q1404001 Q1408039 Q1401107 Q1310019 Q1412045 Q1408035 Q1111033 Q1006051 Q1207025 Q1112013 Q1207049  Q1202023  Q1201023 | |
| Drug | | |  |  | |
| Sulphur Hexafluoride Microbubbles for Injection (Sono Vue contrast) | | | Bracco Suisse SA | J2018005 | |
| Compound Meglumine Diatrizoate Injection | | | Shanghai Xudong Haipu Pharmaceutical Co., Ltd | H31021606 | |
| Oligonucleotides | | |  |  | |
| gRNA sequences for *Pten* see Table S1 | | | This paper; Synthesized by GENEWIZ(Suzhou) | N/A | |
| gRNA sequences for *p53* see Table S1 | | | This paper; Synthesized by GENEWIZ(Suzhou) | N/A | |
| Primers for *Pten* see Table S1 | | | This paper; Synthesized by GENEWIZ(Suzhou) | N/A | |
| Primers for *p53* see Table S1 | | | This paper; Synthesized by GENEWIZ(Suzhou) | N/A | |
| gRNA sequences for Cherry see Table S1 | | | This paper; Synthesized by GENEWIZ(Suzhou) | N/A | |
| Software and Algorithms | | |  |  | |
| Illumina MiSeq Control Software | | | MCS | https://www.illumina.com/systems/sequencing-platforms/miseq.html | |
| Cutadap | | | GENEWIZ | http://www.genewizsz.bioon.com.cn/ | |
| Pandaseq | | | GENEWIZ | http://www.genewizsz.bioon.com.cn/ | |
| Bwa | | | GENEWIZ | http://www.genewizsz.bioon.com.cn/ | |
| Samtools software | | | GENEWIZ | http://www.genewizsz.bioon.com.cn/ | |
| CRISPR design tool | | | Sangsu et al., 2014^47^ | http://www.rgenome.net/cas-offinder/ | |
| BLAST | | | Ladunga, 2017^48^ | http://www.ncbi.nlm.nih.gov/genome/10731 | |
| GraphPad Prism software v 5.0 | | | This paper | http://graphpad-prism.software.informer.com/ | |
| Other | | |  |  | |
| GE Logic E9 | | | GE | USA | |
| GE CT750HD CT | | | GE | USA | |
| SuperCore^TM^ Biopsy Needle MCXS1815LX | | | Argon Medical Inc. | 701118150 | |
| Percutaneous ethanol injection therapy (PEIT) needle | | | Hakko Co., Ltd | 160805 | |
| Laser scanning confocal microscopy | | | Nikon | Nikon AIR | |
| Electrochemiluminescence Immunoassay Instrument | | | Roche. Mannheim | Roche E601 | |
